# Measuring productivity of healthcare services under environmental constraints: evidence from China

**DOI:** 10.1186/s12913-020-05496-9

**Published:** 2020-07-22

**Authors:** Jinna Yu, Zhen Liu, Tingting Zhang, Assem Abu Hatab, Jing Lan

**Affiliations:** 1 Business School, Guizhou Minzu University, Guiyang, 550025 China; 2 School of Business, Nanjing Normal University, No. 1 Wenyuan Road, Nanjing, 210046 China; 3 School of Economics and Management, University of Science and Technology Beijing, Beijing, 100083 China; 4 Department of Economics, Swedish University of Agricultural Sciences, P.O. Box 7013, SE-750 07 Uppsala, Sweden; 5 Department of Economics & Rural Development, Arish University, 45511 Al, Arish, North Sinai Egypt; 6 College of Public Administration, Nanjing Agricultural University, Nanjing, 210095 China

**Keywords:** Healthcare services, TFP, Undesirable output, GML index, Meta-frontier super efficiency SBM model

## Abstract

**Background:**

Despite the growing literature on the efficiency and productivity of the Chinese healthcare system, less attention has been given to examining the undesirable outputs linked to healthcare services, including environmental pollution. Taking the atmospheric environmental pollution resulting from the incineration of medical waste as an undesirable output of the healthcare system, this study analyzed the growth and decomposition of Total Factor Productivity (TFP) of healthcare services across 31 Chinese provinces during the period 2005–2016.

**Methods:**

The Meta-frontier undesirable super-efficiency slack-based measure (SBM) model and the Global Malmquist-Luenberger (GML) Index were employed to analyze the growth and decomposition of TFP using the Max DEA software.

**Results:**

The results revealed that the years 2009 and 2015 marked significant changes in TFP of healthcare services in Chinese provinces. During the study period, the rate of technological change (TC) slowly declined, whereas the rate of efficiency change (EC) steadily increased. With the national average being the benchmark, the results indicated that: the TFP of 17 provinces and cities exceeded the average, the EC of 16 provinces and cities exceeded the average, the TC of 9 provinces and cities exceeded the average, and the value in the Technology Gap Ratio (TGR) changes of 13 provinces and cities were above the national average.

**Conclusions:**

(1) The TFP of the healthcare services across China continued to decline slowly during the study period. (2) The effect of technical catch-up in the eastern, central, and western regions of China was significant across the three regions, whereas the effect of technical innovation was negative. (3) The TFP varied considerably among the Chinese provinces. These findings suggest that, under existing environmental constraints, relevant government departments should improve technical innovation in the supply of healthcare services and medical waste treatment, increase technical efficiency in the factor of healthcare production, strengthen regional health planning, and balance the development of regional healthcare.

## Background

As the negative impact of environmental deterioration on human health continues, green development has become a major target for the international community [1]. Green development emphasizes a harmonious development between human activity and nature. Extensive growth driven only by increasing inputs can no longer satisfy the development of societies. Intensive models of growth, which are driven by improving technical efficiency, are deemed suitable for addressing modern day requirements. The healthcare industry is directly associated with peoples’ physical health. Therefore, it is necessary that it promotes environmental protection. Thus, promoting low energy consumption, low pollution, and low emissions from the healthcare industry should be major objectives for the healthcare industry to achieve green development and enhance the sustainability of healthcare services [2].

In China, hospitals are the main providers of medical services [3]. More efficient medical services can have a positive impact on the development of the entire healthcare system. In this context, issues related to medical waste management have increasingly becoming critical as they pose potential health risks and damage to the environment [4–7]. Specifically, inappropriate management methods exercised during the handling and disposal of medical waste can lead to significant health and environmental hazards [8–10]. The main technical process of the municipal solid waste incineration system in China is the same as that of the medical waste incineration system [11]. According to the “Classified Catalogue of Medical Waste” (2003) and “National Catalogue of Hazardous Waste” (2016) promulgated by China, there are five categories of medical waste, including infectious medical waste, pathological medical waste, traumatic medical waste, drug-induced medical waste and chemical medical waste. Different categories of medical waste have different transfer and disposal processes. The main medical waste incineration technologies include rotary kiln incineration, fixed bed furnace incineration, and pyrolysis incineration [11]. Among them, rotary kiln incineration is the main treatment technology [12]. However, medical waste even after going through these handling and processing procedures can still cause varying degrees of environmental pollution. Therefore, to derive science-based policy recommendations for greening China’s medical and healthcare industry, evaluations of the efficiency and productivity of the medical services should integrate environmental constraints, which may influence the input or output of medical system.

Looking at the medical and healthcare industry as a production unit, hospitals invest human resources, medical equipment, and infrastructure to operate. When the medical system integrates these resources, the medical system provides medical services (mainly outpatient and inpatient services) and it meanwhile produces medical waste. Such undesirable outputs (medical waste) should be considered in assessments of the overall operational efficiency of healthcare services’ providers (e.g. hospitals). Nevertheless, despite the growing literature on the efficiency and productivity of the Chinese healthcare system, less attention has been given to examining the undesirable outputs linked to healthcare services, including environmental pollution. Furthermore, from a policy perspective, equity and efficiency are two factors that constrain healthcare outputs (i.e. the health level) in China, with the most important priority right now being the improvement of efficiency. On the one hand, such a demand is a requirement of supply-side reforms in the medical and healthcare industry. On the other, with the emergence of universal health insurance coverage, the equity issue in the medical system has improved, which implies that the next focus area of medical reform should be the improvement of efficiency [13]. Concurrently achieving efficiency and equity has been the purpose of governments of all nations. However, there exists a symbiotic relationship between efficiency and equity. As efficiency is a material prerequisite for fairness, ignoring efficiency not only hinders the realization of equity (regardless of the amount of the health inputs), but may also cause the waste of scarce healthcare resources, which is not conducive to the development of China’s medical and healthcare industry.

Against this background, this paper uses the Meta-frontier undesirable super-efficiency slack-based measure (SBM) model and the Global Malmquist-Luenberger (GML) Index to analyze the growth and decomposition of healthcare services’ TFP in Chinese provinces during the period 2005–2016. The empirical analysis was carried out using the MaxDEA software and relied on a balanced panel dataset, from China Health and Family Planning Statistical Yearbook, covering the 31 Chinese provinces for the period 2005–2016 was used (*n* = 372). This paper adds to the literature in several important ways. First, although several studies have used the meta-frontier non-radial Malmquist index method to analyze the TFP of different sectors, such as banking sector [14] and fossil fuel power plants [15], the method has not been used in analyses focusing on the healthcare sector. Second, unlike the traditional data envelope analysis method which follows the radial principle when making efficiency calculations and improves inputs and outputs proportionally, this study considers both the desirable output and the undesirable output and thus it adopts a non-radial slack-based measure (SBM) model. Moreover, the super efficiency model was used to avoid decision-making units (DMUs) from being DEA efficient but unable to be sorted. Third, the heterogeneity in social and economic development among China’s eastern, central, and western regions implies that ignoring the technical heterogeneity among these regions can lead to misleading and inaccurate calculations of healthcare efficiency. Therefore, this paper uses the Meta-frontier method which constructs different group frontiers according to technical heterogeneity and measures the gap between different research objects, which considers the factors influencing technical heterogeneity in the calculation of efficiency scores.

## Data and research methods

### Data sources

The data set used in this study came from the 2006–2017^1^ China Health and Family Planning Statistical Yearbook. Using the carbon emission coefficient from the 2006 IPCC National Greenhouse Gas Inventory Guide and different discharge coefficients of medical waste after disposal from the Chinese Manual of Pollutant Generation and Emission Factors in Hospitals, we calculated undesirable outputs. Given that this study uses panel data, value indicators from all the years were converted to the 2005 price level to facilitate direct comparisons. In addition, the division of the eastern, central, and western regions mentioned was based on the classification of National Bureau of Statistics and included 11 provinces and cities in the eastern region, eight provinces and cities in the central region, and 12 provinces and cities in the western region.^2^

### Selection of variables

In conjunction with existing literature [16], input indicators consist of healthcare personnel and fixed assets in hospitals. Output indicators consist of the total number of outpatient clinics and admissions provided by hospitals.^3^ Data on healthcare personnel, the total number of outpatient consultations and admissions were collected from the Health Statistics Yearbook. Fixed assets were calculated using the proportion of beds in hospitals and township healthcare centers. The value of such fixed assets was converted using the fixed asset value index.

Undesirable output indicators consist of CO_2_, COD, and NH_3_-N. CO_2_ was used to measure atmospheric environmental pollution resulting from the incineration of medical waste. Specifically, COD and NH_3_-N were used to measure the organic and inorganic water pollution resulting from the treatment of sewage produced during the provision of medical services.^4^ To calculate such undesirable outputs, the total amount of medical waste and sewage produced by a hospital was calculated according to its pollutant emission coefficient. Then, the CO_2_ produced by the incineration of medical waste was calculated according to the IPCC National Greenhouse Gas Inventory Guide. Eventually, the COD and NH_3_-N generated by sewage discharge were calculated according to the pollutant emission coefficient.^5^

### Data envelope analysis

Research related to efficiency commonly uses the data envelope analysis (DEA) and Stochastic Frontier Analysis (SFA) [17]. There are other methods that can be used, such as the RSR method (Rank Sum Ratio), the TOPSIS method (Technique for Order Preference by Similarity to Ideal Solution) [18], comprehensive evaluation index method, and ratio analysis method. However, there is little research that uses these methods because of their limitations [19]. The DEA method was introduced into the medical field by Sherman [20]. Since then, the use of DEA methods to evaluate the efficiency of medical services has increased rapidly [21]. For example, the DEA approach is often used to evaluate changes in hospital efficiency, usually as a result of medical service reforms [22, 23]. In addition, research subjects may be found at varying macro- and micro-levels, such as the OECD National Health System [24] and various other health projects [25] and doctors [26]. In recent years, DEA has been increasingly adopted in analyses focusing on the medical and health-related fields. For example, the super-efficiency DEA was used to explore the efficiency of acute care hospitals in Pennsylvania [27], and the network DEA applications were used in the healthcare systems [28]. In addition, other researchers introduced the Bootstrap DEA approach [29, 30], Cross-efficiency DEA approach [31], Multi-stage DEA model [32] and Multifactor efficiency DEA [33] into healthcare research.

Despite the extensive use of DEA in the evaluation of healthcare efficiency, several studies have pointed out its theoretical and methodological limitations. To address these limitations, a common strategy has been to combine DEA with other methods and techniques to provide better efficiency evaluation [16]. For example, various DEA models have been employed together with Malmquist indices and econometric models to evaluate inefficiency in addition to measuring efficiency, such as DEA-Malmquist-Tobit model [34], DEA-Tobit model [35, 36], DEA-Malmquist model [37].

The DEA analysis of medical service efficiency in China is mainly focused on studying technical efficiency and productivity. The models used are relatively simple, including the traditional DEA and MI models [38, 39]. Although some scholars tried to use the DEA extension model, the studies remain very few compared to those that adopted the traditional DEA model. Other scholars have combined a variety of DEA extension models with MI and other econometric models, such as Tobit [13, 40].

In terms of research subjects, there are not only international examples, but also Chinese hospitals at the inter-provincial level, the municipal level, and other levels. Regarding the selection of research indicators, existing literature is mostly concerned with the input constraints of human and material resources, even though some scholars consider undesirable outputs in their studies. For example, as hospital operations produce undesirable outputs like patient mortality, which may induce medical disputes and litigations, Hu et al. [41] adopted DEA to assess and compare the efficiency scores obtained with and without the undesirable output.

### Meta-US-SBM model

In existing research, when the DEA method is used to study efficiency in healthcare, some scholars use an input orientation, while others use an output orientation. The efficiency in this paper means to use as little medical resources as possible to maximize expected outputs and minimize undesirable outputs. However, the traditional DEA model was used to improve efficiency attempts and thus reduce all inputs (outputs) proportionally and radially. Therefore, a non-oriented non-radial SBM model was used to achieve the objectives of this study. To sort the efficiency of research objects, the super efficiency model was chosen. In addition, considering the regional differences in China’s healthcare industry, Meta-frontier and group frontier were used to measure efficiency. Because panel data was used in this study, the Malmquist productivity index was used to study the spatial and temporal changes of medical service efficiency and productivity in China. In summary, this study used the Meta-frontier undesirable super efficiency slack-based measure model and the Global Malmquist-Luenberger index model, which together can be stated as the META-US-SBM-GML model.

Supposing that there are *N* DMUs in the study (31 provinces) which belong to different technical heterogeneous groups *H* (this article refers to the eastern, central and western regions of China), the group *h* has *N*_*h*_ DMUs, where it is assumed ${\sum}_{h=1}^H{N}_h=N$. Each DMU has *m* inputs, *q*_1_ desirable outputs, and *q*_2_ kinds of undesirable outputs. The input and output variables for each DMU are $X=\left({x}_1,{x}_2,\cdots, {x}_m\right)\in {R}_m^{+}$, $Y=\left({y}_1,{y}_2,\cdots, {y}_{q_1}\right)\in {R}_{q_1}^{+}$, and $B=\left({b}_1,{b}_2,\cdots, {b}_{q_2}\right)\in {R}_{q_2}^{+}$, respectively, where *X* > 0, *Y* > 0, *B* > 0 are assumed. The frontier of production technology for group *h* can be expressed as:

1 $$ {P}^h=\left\{\left({x}^h,{y}^h,{b}^h\right):\sum \limits_{n=1}^{N_h}{\lambda}_n^h{x}_n^h\le {x}^h;\sum \limits_{n=1}^{N_h}{\lambda}_n^h{y}_n^h\ge {y}^h;\sum \limits_{n=1}^{N_h}{\lambda}_n^h{b}_n^h\le {b}^h;n=1,2,\cdots, {N}_h\right\} $$

where ${\lambda}_n^h$ is the weight vector of the production technology frontier of the *n*^th^ DMU reference in group *h*. Meta-frontier technology is the envelope line at the forefront of production technology for all groups, which can be expressed as:

2 $$ {P}^{meta}=\left\{\begin{array}{l}\left(x,y,b\right):\sum \limits_{h=1}^H\sum \limits_{n=1}^{N_h}{\xi}_n^h{x}_n^h\le {x}^h;\sum \limits_{h=1}^H\sum \limits_{n=1}^{N_h}{\xi}_n^h{y}_n^h\ge {y}^h;\sum \limits_{h=1}^H\sum \limits_{n=1}^{N_h}{\xi}_n^h{b}_n^h\le {b}^h;\\ {}n=1,2,\cdots, {N}_h;h=1,2,\cdots, H\end{array}\right\} $$

where *P*^*meta*^ = {*P*^1^ ∪ *P*^2^ ∪ ⋯ ∪ *P*^*H*^}; ${\xi}_n^h$ is the weight vector of the *n*^th^ DMU in group *h* with reference to Meta-frontier technology *P*^*meta*^. Using the definition of the frontier of group production technology and Meta-frontier technology, non-oriented super efficiency SBM can be defined based on two different frontiers. Assuming that the return of scale remains the same, the optimal goal of the *o*^th^ DMU of the *k*^th^ group, based on the frontier of group production technology, is (*o* = 1, 2, ⋯, *N*_*k*_, *k* = 1, 2, ⋯, *H*):

3 $$ {\displaystyle \begin{array}{l}{\rho}_{ko}^{group\ast }=\min \left(\frac{1+\frac{1}{m}\sum \limits_{i=1}^m{s}_{iko}^{-}/{x}_{iko}}{1-\frac{1}{q_1+{q}_2}\left(\sum \limits_{r=1}^{q_1}{s}_{rko}^{+}/{y}_{rko}+\sum \limits_{j=1}^{q_2}{s}_{jko}^{b-}/{b}_{jko}\right)}\right)\\ {}s.t.\kern0.5em \sum \limits_{n=1,n\ne o}^{N_k}{\lambda}_n^k{x}_{ikn}-{s}_{iko}^{-}\le {x}_{iko}\\ {}\kern1.00em \sum \limits_{n=1,n\ne o}^{N_k}{\lambda}_n^k{y}_{rkn}+{s}_{rko}^{+}\ge {y}_{rko}\\ {}\begin{array}{ccc}& & \sum \limits_{n=1,n\ne o}^{N_k}{\lambda}_n^k{b}_{jkn}-{s}_{jko}^{b-}\le {b}_{jko}\end{array}\\ {}\begin{array}{ccc}& & 1-\frac{1}{q_1+{q}_2}\left(\sum \limits_{r=1}^{q_1}{s}_{rko}^{+}/{y}_{rko}+\sum \limits_{j=1}^{q_2}{s}_{jko}^{b-}/{b}_{jko}\right)\ge \varepsilon \end{array}\\ {}\begin{array}{ccc}& & {\lambda}_n^k,{s}_{iko}^{-},{s}_{rko}^{+},{s}_{jko}^{b-}\ge \end{array}0\\ {}\begin{array}{ccc}& & i=1,2,\cdots, m;\begin{array}{c}\end{array}r=1,2,\cdots, {q}_1;\begin{array}{c}\end{array}j=1,2,\cdots, {q}_2\end{array}\end{array}} $$

If a constraint condition ${\sum}_{n=1,n\ne o}^{N_k}{\lambda}_n^k=1$ is added to formula (3), it represents the non-oriented super efficiency SBM model in the *o*^th^ DMU in group *k* based on the frontier of group production technology under the conditions of variable return of scale (VRS). Similarly, under the conditions of constant return of scale (CRS), the optimal goal of the *o*^th^ DMU in group *k*, based on Meta-frontier technology, is (*o* = 1, 2, ⋯, *N*_*k*_ , *k* = 1, 2, ⋯, *H*):

4 $$ {\displaystyle \begin{array}{l}{\rho}_{ko}^{meta\ast }=\min \left(\frac{1+\frac{1}{m}\sum \limits_{i=1}^m{s}_{iko}^{-}/{x}_{iko}}{1-\frac{1}{q_1+{q}_2}\left(\sum \limits_{r=1}^{q_1}{s}_{rko}^{+}/{y}_{rko}+\sum \limits_{j=1}^{q_2}{s}_{jko}^{b-}/{b}_{jko}\right)}\right)\\ {}s.t.\kern0.5em \sum \limits_{h=1}^H\sum \limits_{\begin{array}{l}n=1,n\ne o\\ {} if\begin{array}{c}\end{array}h=k\end{array}}^{N_k}{\xi}_n^h{x}_{ihn}-{s}_{iko}^{-}\le {x}_{iko}\\ {}\begin{array}{ccc}& & \sum \limits_{h=1}^H\sum \limits_{\begin{array}{l}n=1,n\ne o\\ {} if\begin{array}{c}h=k\end{array}\end{array}}^{N_k}{\xi}_n^h{y}_{rhn}+{s}_{rko}^{+}\ge {y}_{rko}\end{array}\\ {}\begin{array}{ccc}& & \sum \limits_{h=1}^H\sum \limits_{\begin{array}{l}n=1,n\ne o\\ {} if\begin{array}{c}\end{array}h=k\end{array}}^{N_k}{\xi}_n^h{b}_{jhn}-{s}_{jko}^{b-}\le {b}_{jko}\end{array}\\ {}\begin{array}{ccc}& & 1-\frac{1}{q_1+{q}_2}\left(\sum \limits_{r=1}^{q_1}{s}_{rko}^{+}/{y}_{rko}+\sum \limits_{j=1}^{q_2}{s}_{jko}^{b-}/{b}_{jko}\right)\ge \varepsilon \end{array}\\ {}\begin{array}{ccc}& & {\xi}_n^h,{s}_{iko}^{-},{s}_{rko}^{+},{s}_{jko}^{b-}\ge 0\end{array}\\ {}\begin{array}{ccc}& & i=1,2,\cdots, m;\begin{array}{c}\end{array}r=1,2,\cdots, {q}_1;\begin{array}{c}\end{array}j=1,2,\cdots, {q}_2\end{array}\end{array}} $$

The above model is under the assumptions CRS income. If it was under the assumption of VRS, the constraint condition $$ \sum \limits_{h=1}^H\sum \limits_{\begin{array}{l}n=1,n\ne o\\ {} if\kern0.5em h=k\end{array}}^{N_k}{\xi}_n^h=1 $$ needs to be added. In formula (3) and formula (4), ${s}_{iko}^{-},{s}_{rko}^{+},{s}_{jko}^{b-}$ represent the slack variables of input, desirable output, and undesirable output, respectively. *ε* indicates the non-Archimedes infinitesimal. The constraint is to ensure that the denominator of the objective function is greater than 0.

### Meta-frontier-global-Malmquist-Luenberger index

To analyze the effect of changes in DMU productivity, technical efficiency, and technological changes in productivity, it is necessary to use the Malmquist (M) Index. Färe et al. [42] calculated the M index for the first time using the DEA method and decomposed the M index into changes in technological efficiency (TE) and technological change (TC). Chung et al. [43] applied the directional distance function containing undesirable outputs to the M index model. Then, scholars began to refer to the M index, which contains undesirable outputs, as well as the ML index. Oh and Lee [44] applied the Meta-frontier method to the global reference M index and decomposed Meta-M. Following existing studies, the decomposition process of the META-GML used in this study is shown in formula (5):

5 $$ {\displaystyle \begin{array}{l}{ML}_g^{meta}\left({x}^{t+1},{y}^{t+1},{b}^{t+1},{x}^t,{y}^t,{b}^t\right)=\frac{\rho_g^{meta}\left({x}^{t+1},{y}^{t+1},{b}^{t+1}\right)}{\rho_g^{meta}\left({x}^t,{y}^t,{b}^t\right)}\\ {}=\frac{\rho_{t+1}^{group}\left({x}^{t+1},{y}^{t+1},{b}^{t+1}\right)}{\rho_t^{group}\left({x}^t,{y}^t,{b}^t\right)}\left(\frac{\frac{\rho_g^{meta}\left({x}^{t+1},{y}^{t+1},{b}^{t+1}\right)}{\rho_{t+1}^{group}\left({x}^{t+1},{y}^{t+1},{b}^{t+1}\right)}}{\frac{\rho_g^{meta}\left({x}^t,{y}^t,{b}^t\right)}{\rho_t^{group}\left({x}^t,{y}^t,{b}^t\right)}}\right)\\ {}=\frac{\rho_{t+1}^{group}\left({x}^{t+1},{y}^{t+1},{b}^{t+1}\right)}{\rho_t^{group}\left({x}^t,{y}^t,{b}^t\right)}\left(\frac{\frac{\rho_g^{group}\left({x}^{t+1},{y}^{t+1},{b}^{t+1}\right)}{\rho_{t+1}^{group}\left({x}^{t+1},{y}^{t+1},{b}^{t+1}\right)}}{\frac{\rho_g^{group}\left({x}^t,{y}^t,{b}^t\right)}{\rho_t^{group}\left({x}^t,{y}^t,{b}^t\right)}}\right)\left(\frac{\frac{\rho_g^{meta}\left({x}^{t+1},{y}^{t+1},{b}^{t+1}\right)}{\rho_g^{group}\left({x}^{t+1},{y}^{t+1},{b}^{t+1}\right)}}{\frac{\rho_g^{meta}\left({x}^t,{y}^t,{b}^t\right)}{\rho_g^{group}\left({x}^t,{y}^t,{b}^t\right)}}\right)\\ {}=\frac{TE_{t+1}^{group}}{TE_t^{group}}\left(\frac{BPG_{t+1}^{group}}{BPG_t^{group}}\right)\left(\frac{TGR^{t+1}}{TGR^t}\right)\\ {}= EC\ast BPC\ast TGC\end{array}} $$

where, EC refers to the change of *TE* within each group from period t to period t + 1; BPG (Best Practice Gap) refers to the gap between the current frontier of each group and the global frontier of each group^6^; BPC (Best Practice Change) refers to the changes in the BPG from period t to period t + 1; TGR refers to the gap between the global frontier and the Meta-frontier of each group; TGC is the change in the TGR that occurs from period t to period t + 1. The EC is regarded as the “catch-up effect” because it can capture the closeness to the current frontier in period t + 1 of the decision-making unit relative to period t. If EC > 1, it indicates improvements in efficiency; the opposite indicates a reduction in efficiency. BPC indicates the extent to which the current frontier within the group moves to the global frontier of the group, which is to say the degree of technological change. As technological innovation has the potential to move the production frontier forward, it can be regarded as the “innovation effect”. If BPC > 1, it means that the frontier of group production technology is close to the global frontier of the group and is tending toward producing more expected outputs and fewer undesirable outputs. On the contrary, if BPC < 1 it means that the current frontier of the group is far from the global frontier of the group. TGC measures the degree of change in the technological gap between the meta-frontier and the global frontier of the group across two periods, which is regarded as the “leading effect”. If TGC > 1, it means that the gap between the research object and Meta-frontier technology is narrowing, which is to say the research object, in terms of technology, is in a lead position among the other research objects. Otherwise, the research object is in a technologically backward position.

## Results

### Descriptive analysis of input-output indicators

The descriptive statistics of the input-output variables are detailed in Table 1. According to the coefficient of variation, the degree of dispersion of each variable is generally the same (all around 0.7), with only the total number of outpatient consultations showing fluctuation, at 0.869.

**Table 1:** Descriptive statistics of variables

Indicator		symbol	Unit	Sample size	Minimum	maximum	Mean	standard deviation	variation coefficient
Input	Healthcare personnel	*HW*	pPerson	372	8064	636,575	196,693	130,381	0.663
	Fixed assets	*Cap*	Million yuan	372	86,857	12,797,953	3,243,027	2,512,375	0.775
Output	Total number of outpatient consultations	*OP*	Person-time	372	4,612,351	551,857,596	116,626,362	101,404,835	0.869
	Number of admissions	*IP*	Person-time	372	78,764	15,951,005	4,590,540	3,560,248	0.776
	Carbon dioxide emissions	*CO*_*2*_	*Gg*	372	0.363	34.713	10.659	7.332	0.688
	Chemical Oxygen Demand	*COD*	Tons	372	148	13,495	4080	2797	0.686
	Ammonia nitrogen	*NH*_*3*_*-N*	Tons	372	20	1998	618	425	0.688

GG is a unit of CO_2_ emissions measured in the IPCC climate list, 1GG = 1000 tons

Because the data used in this study are panel data, it is necessary to conduct a descriptive statistical analysis of the relative values, in addition to conducting descriptive analysis of the absolute values. In particular, the growth rate of each input-output variable and the share of undesirable outputs are depicted in Table 2.

**Table 2:** 2005–2016 Growth rate of variables in eastern, central, and western China and their share of pollutant emissions(%)

Region	Average growth rate							Share of emissions		
	*HW*	*Cap*	*OP*	*IP*	*CO*_*2*_	*COD*	*NH*_*3*_*-N*	*CO*_*2*_	*COD*	*NH*_*3*_*-N*
Eastern average	6.44	9.32	8.35	9.82	6.76	6.79	6.93	41.04	41.14	41.45
Central average	5.29	13.12	7.47	11.63	8.03	8.06	8.15	31.26	31.23	31.01
Western average	7.68	13.17	7.49	12.64	8.91	8.88	9.01	27.70	27.62	27.54
National average	6.40	11.09	7.94	11.16	7.74	7.75	7.87	100	100	100

As the calculation results are rounded, the emissions across eastern, central and western China do not equal 100%

Regarding inputs, the growth rates for capital inputs nationally and across each of the three regions are higher than those for human resources. In particular, the western region grew the fastest in terms of both human resources and capital inputs. Concerning outputs, the growth rate of inpatient services is higher than that of outpatient services nationally and across each region, while the three kinds of undesirable outputs have roughly the same growth rates. The fastest growth in inpatient and outpatient services appears in the western and eastern regions, with the western region being the fastest growing area for undesirable outputs of medical services. Emissions from undesirable outputs in the eastern region far exceed the central and western regions, with the western region having the lowest share of undesirable outputs. In addition to the number of outpatient services, the growth rates for the remaining input-output variables in the western region are the highest of the three regions, which are above the national average. However, the share of pollutant emissions in the western region is the lowest among the three regions. The growth rate for inputs and outputs in the provision of medical services suggest that, since the growth rate of capital input is the highest among all input-output variables, the growth rate of inpatient services ranks second, with the average annual growth rate of each exceeding 10%. The growth rate of both human resources and outpatient services lags far behind that of capital and inpatient services, both below the average annual growth rate of 8%. Unbalanced structural growth can affect productivity growth.

### The evolution of TFP change and decomposition of medical services in China annually

**Fig. 1:**
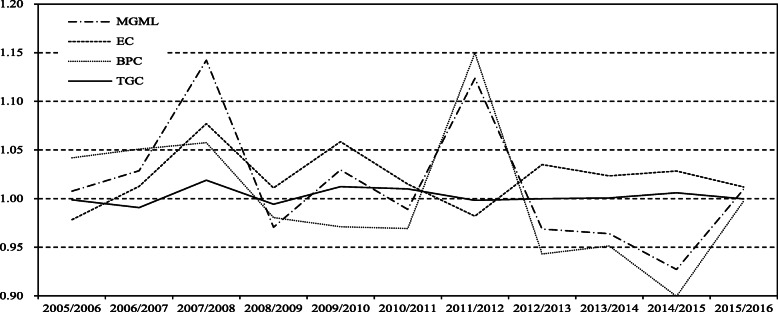
TFP change and the decomposition effect of medical services in China in 2005–2016

Figure 1 depicts a downward trend in TFP in the provision of medical services in China. The first valley appeared in 2009, which was the year of China’s new medical reforms. In 2009, many policies were issued, which started with the reform of medical and healthcare institutions and were to solve the dual issues of medical treatment being inaccessible and expensive. Therefore, driven by top-down policies, the reorganization of the healthcare system’s resources, including the merger and restructuring of institutions, emphasized public welfare in hospitals and capacity building in primary medical care clinics. Strong grassroots policies were to support the provision of basic medical hardware. Although human resources support was issued, the training of talent lagged far behind capital inputs, which led to a slight decline in the level of productivity in healthcare services.

The second valley of TFP occurred in 2015, when the State emphasized a healthy China strategy which put forward higher requirements for the medical service system. The strategy required medical services to satisfy residents’ need for personalized medical services while providing them with basic medical services. Meanwhile, the strategy required reasonable medical cost controls. This represented a significant challenge for hospitals, which are the core of the healthcare system. In addition, it instituted stringent requirements for environmental protection. If medical institutions do not do well in environmental protection, the polluted environment can affect the health of residents. Consequently, the workload and burden of medical institutions are exacerbated, resulting in a downward trend in total factor productivity, which forms a vicious circle. Therefore, in order to achieve a continuous growth trend of total factor productivity of medical services in China, while strengthening the progress of medical technology, it is necessary to strengthen the research and development of technologies for medical waste treatment, so that medical waste can be released into the environment without toxic and harm after treatment. A fundamental driver behind the growth of total factor productivity in medical services is to increase the output of medical services while reducing the pollution of medical waste.

Although EC has a decline trend, it is bigger than 1 except for the year 2012, which means the progress of technological efficiency of medical system in China, thus the catch-up effect is obvious with time flies. The TGC shows a slow growth trend. Especially after the new medical reforms, the TGC shows a clear growth trend. Such results suggest that the gap between the global technology frontier of the three regions and the Meta-frontier for technology in China is gradually narrowing. BPC which means innovation effect experiences strong fluctuation in 2012 and 2015, respectively. Especially, during two periods 2008–2010 and 2012–2015, BPC has sharp decline trend, which shows weaken of innovation effect of medical system in China. Figure 1 also shows that the TFP change of medical system is mainly depend on the catch-up effect before the year 2011, and then is mainly depend on the innovation effect.

### TFP change and the decomposition effect of medical services in various regions of China

Given its economic development and geographical location, China can be divided into three regions, namely the eastern, central and western regions. Based on the methodology presented in Section 2 of this paper, the Meta-frontier GML index captures the change of productivity of medical services, thus the scores of GML index indicates the increased or decreased change of TFP of the medical services. The decomposition of the Meta-frontier GML index can identify technological efficiency change within a group (EC), changes in the differences between the current frontier within each group and the global frontier (BPC), and the ratio of changes in the differences between the global frontier of each group and the Meta-frontier (TGC). This indicates the presence of a catch-up effect, innovation effect, and leading effect as regards the research object, respectively. The results are displayed in Table 3.

**Table 3:** TFP change and the decomposition effect of medical services in China and its sub-regions

Time	Eastern region				Middle region				Western region			
	MGML	EC	BPC	TGC	MGML	EC	BPC	TGC	MGML	EC	BPC	TGC
2005/2006	0.9198	0.9599	0.8657	0.9240	0.9178	0.8607	1.0669	0.9916	1.0630	0.9865	1.1057	0.9886
2006/2007	1.0765	0.9627	1.1416	0.9972	1.0044	1.0660	0.9552	1.0024	1.0011	1.0231	1.0314	0.9774
2007/2008	1.0683	1.1337	1.0009	0.9642	1.1630	1.0441	1.0305	1.0921	1.1962	1.0470	1.1275	1.0201
2008/2009	0.9737	1.0580	0.9103	1.0276	0.9669	1.0424	0.9474	0.9832	0.9700	0.9469	1.0672	0.9711
2009/2010	1.0608	1.0059	1.0647	0.9917	1.0109	1.0296	0.9956	0.9878	1.0135	1.1260	0.8689	1.0476
2010/2011	1.0235	1.0022	1.0155	1.0155	0.9676	1.0014	0.9515	1.0196	0.9715	1.0358	0.9386	0.9986
2011/2012	1.1401	0.9592	1.2235	0.9775	1.0992	0.9982	1.1050	0.9955	1.1247	0.9922	1.1119	1.0191
2012/2013	0.9906	1.0718	0.9243	1.0200	0.9881	1.0582	0.9544	0.9795	0.9358	0.9859	0.9530	0.9948
2013/2014	0.9902	1.0170	0.9930	1.0044	0.9752	1.0220	0.9600	0.9999	0.9324	1.0302	0.9075	0.9977
2014/2015	0.9270	1.0557	0.8815	1.0012	0.9297	0.9979	0.9167	1.0201	0.9254	1.0236	0.9046	1.0010
2015/2016	1.0496	1.0163	1.0344	0.9965	0.9989	1.0188	0.9760	1.0049	0.9802	1.0034	0.9780	0.9994
Average	1.0180	1.0208	0.9997	0.9923	0.9998	1.0112	0.9857	1.0065	1.0072	1.0173	0.9955	1.0012

First, the average annual growth rate of TFP, EC, BPC, and TGC in eastern China is 1.8, 2.08, − 0.03%, and − 0.77%, respectively. These results indicate that the growth of TFP in the eastern region is largely influenced by the catch-up effect, while the innovation effect and leading effect are insignificant. The average annual growth rate of TFP, EC, BPC, and TGC in the central region is −0.02, 1.12, −1.43%, and 0.65%, respectively. These results indicate that the decline of TFP in the central region is restricted by the effect of technological innovation. In addition, the catch-up effect is the main factor slowing down the decline of the TFP change. However, the leading effect is not significant. The average annual growth rate of TFP, EC, BPC, and TGC in western China is 0.72, 1.73, − 0.45%, and 0.12%, respectively. These results imply that the growth of TFP in the western region relies on the catch-up effect, while the innovation effect is not significant. Thus, the growth of TFP in the three regions of China is slow. The central region in particular has shown a decline each year. The western region is between the central and eastern regions.

In terms of the decomposition effect, the growth of TFP in China’s three regions still depends on the catch-up effect, while technological innovation and the leading effect generally restrain the growth of TFP. This means that currently the medical field in China still relies on increasing the input of health resources, rather than on technological innovation and advances, to move closer to the technological frontier. Because the TGR in the central region is growing the fastest, it is dominating the technological frontier in China’s medical services sector. Such results seem to contradict the prevailing idea that medical services in the eastern region are better than those in the central and western regions. Such a contradictive result may be because this study considers environmental constraints. The growth rate of inputs and outputs in the central region is in-between that of the eastern and western regions; its proportion of emissions is higher than the western region and lower than the eastern region. However, the service volume in absolute terms in the central region is no less than that in the eastern and western regions. Since outpatient and inpatient services are the main output indicators in this study, the absolute service volume may be the reason for the central region leading the others in terms of technology.

Second, from a temporal perspective, the results show that the TFP growth in the three regions fluctuated over time. The highest growth rate in the central and western regions was seen before the new medical reforms, while the highest growth rate in the eastern region occurred after the new medical reforms. The growth rate in the eastern region is significantly higher than that in the central and western regions after the new medical reforms. This is because the eastern region made significant innovations to promote the development of the medical industry during the reform period. However, the central and western regions are not as advanced, due to their economic and geographic disadvantages. However, the three regions have shown high growth rates since 2015. Given the time lag in policy development and implementation, this may be due to the effect of China’s new healthcare reforms.

Considering the decomposition effect, changes in technical efficiency show a steady upward trend. This is probably because the Chinese government is paying special attention to the development of the medical system and is increasing the intensity of medical inputs, a process which drives medical institutions to increase their input factors to obtain gains in efficiency. However, technological change shows a downward trend. An explanation could be that environmental constraints create high requirements for technological innovation and advances in healthcare institutions, especially with regard to the generation, treatment, and release of medical waste. Obviously, the data demonstrates that there has since 2015 been a rebound in technological change, which is related to State Health Administration policies which stress the treatment of medical waste. The results also indicate that the green development theory seems to have been integrated into the production and operational processes of medical institutions, causing technological change to increase.

According to the change of TGR, even though the eastern region is leading in terms of growth rate, the gap between its technology frontier and China’s Meta-frontier for technology is the smallest. However, there is a slow decline in the overall trend; its highest growth rate appeared in 2009. The change in the TGR in the eastern and western regions shows an increasing trend, with the growth rate of the central and eastern regions being higher than that of the western region. The TGRs of the three regions were all similar in 2016. The TGR in the three regions shows convergence over time, which suggests that because of the reforms and overall development of the medical service industry, the TFP gap caused by regional technical differences is narrowing.

To sum up, the medical and healthcare industry’s technological progress across all three regions has regressed. The growth of TFP in the eastern, central, and western regions depends on the improvement of technological efficiency, while being constrained by technological change. In addition, the TGR caused by technological heterogeneity among three regions shows convergence over time.

### Decomposition effect of TFP change in the provision of medical services in Chinese provinces

As Table 4 shows, there are important differences in the TFP and decomposition among provincial medical services. With the national average being the benchmark, there are 17 provinces and cities that have growths of TFP exceeding the average, 16 provinces and cities that have EC above average, 9 provinces and cities that have TC exceeding the average, and 13 provinces and cities that have changes in the TGR above average. Due to the consideration of environmental constraints, the provinces with high TFP changes include most of the eastern and central regions, as well as a few western regions. The provinces and cities experiencing significant technological catch-up effects are located in the eastern and central regions, in cities such as Beijing, Tianjin, and Shanghai in the east, Henan and Hunan in the central region, and Yunnan and Shaanxi in the west. The provinces experiencing the technological innovation effect are primarily located in the eastern region, with only three provinces from the central and western regions, namely, Hubei, Tibet, and Xinjiang. This shows that technological change in the medical field is largely seen in the eastern region. Given that the eastern region has geographical advantages and opened up earlier, it has greater innovation than the central and western regions. The provinces with relatively high growth in TGRs are mainly located in the eastern and central regions, with only three provinces in the western region, namely, Chongqing, Sichuan, and Gansu provinces. These provinces are thus closer to the Meta-frontier of China as a whole. In provinces such as Inner Mongolia, Jilin, Guangxi, Guizhou, Qinghai, and Ningxia, which are located in western China, four indicators are below the national average. Specifically, TFP changes, changes in technological efficiency, technological changes, and changes in the TGR have negative growth. Although these regions have governmental policy support, which resulted in a substantial increase in health inputs, there is a lack of human resources due to weaknesses in their economic and geographic conditions. This means that a lack of human resources relative to material health resources can lead to the inefficiencies in the provision of medical services and hinder development.

**Table 4:** TFP change and the decomposition effect of medical services in China’s provinces, 2005–2016

Provinces	MGML	EC	BPC	TGC	Provinces	MGML	EC	BPC	TGC
Beijing	1.1071	1.0806	1.0245	1.0001	Hubei	1.0270	1.0008	1.0152	1.0108
Tianjin	1.0470	1.0361	1.0089	1.0016	Hunan	1.0183	1.0447	0.9734	1.0014
Hebei	1.0091	1.0006	1.0167	0.9919	Guangdong	1.0055	0.9978	1.0094	0.9983
Shanxi	1.0130	1.0222	0.9821	1.0090	Guangxi	0.9928	1.0031	0.9890	1.0007
Inner Mongolia	0.9824	0.9934	0.9919	0.9970	Hainan	0.9905	0.9999	0.9892	1.0014
Liaoning	1.0235	1.0402	0.9862	0.9977	Chongqing	0.9861	1.0131	0.9699	1.0035
Jilin	0.8886	0.9170	0.9799	0.9889	Sichuan	0.9881	1.0008	0.9849	1.0024
Heilongjiang	1.0160	1.0258	0.9850	1.0056	Guizhou	0.9901	0.9950	0.9958	0.9993
Shanghai	1.0511	1.0261	1.0244	1.0000	Yunnan	1.0099	1.0204	0.9899	0.9998
Jiangsu	1.0099	1.0242	0.9865	0.9995	Tibet	1.0001	1.0012	1.0008	0.9981
Zhejiang	1.0270	1.0175	1.0092	1.0001	Shaanxi	1.0051	1.0206	0.9861	0.9988
Anhui	0.9896	1.0063	0.9771	1.0065	Gansu	1.0213	1.0306	0.9896	1.0013
Fujian	0.9807	0.9910	0.9746	1.0154	Qinghai	0.9805	0.9910	0.9917	0.9976
Jiangxi	0.9868	0.9989	0.9777	1.0103	Ningxia	0.9879	1.0086	0.9837	0.9957
Shandong	1.0133	1.0351	0.9874	0.9915	Xinjiang	1.0187	1.0156	1.0028	1.0002
Henan	1.0002	1.0159	0.9809	1.0036					
National	**1.0048**	**1.0117**	**0.9923**	**1.0009**					

## Discussion

The three regions in China feature different socio-economic environments [3], and are in the different position of national health system plans, resulting in disparate demand and supply of health services, which will lead to different operation efficiency and productivity. Besides, the different healthcare inputs and outputs of three regions are probably closely related to national support policies. The State is working to increase the inputs for the medical and healthcare industry in the western region. The western provinces and municipalities are also working to construct infrastructure for medical institutions. As a result, the growth rate of fixed assets and health-related human resources in the western region is the highest among the three regions, indicating regular improvements of its medical service system. Liu et al. [45] also mentioned the imbalance in the allocation of health resources, in their study, they found that eastern region had better health resources.

In this paper, the growth rates for capital inputs in China and three regions are higher than those for human resources, implying that the health system of China and three regions had experienced the higher scale development and less capacity development in the 2005–2016 period. This is same with the results of Li et al. [46], in their study, they also found that the growth rates of the fixed asset (capital) is highest of all inputs. For outputs, the growth rate of inpatient services is higher than that of outpatient services in China and three regions, while the three kinds of undesirable outputs have roughly the same growth rates. This is different with the results of Sun and Luo [47], in their study, they found that eastern region provided more outpatient care, while western region provided more inpatient care. In Ding et al. [48] study, they showed that the eastern region allocated more human and capital resources than the other two regions, whereas the western region produced more inpatient services.

This study found that the TFP change of China and its three regions has the similarly alternative trend of increase and decrease after 2009. The TFP change of China medical services experience regular fluctuation, take the year 2009 as turning point, it is obvious that the decreasing trend of sine wave both before and after 2009, but the latter is lower than before. It means that when we consider the environmental limit, nowadays, the TFP of medical services is not as higher as that of before, on the other hand, it means there is still room to improve for the TFP of medical services. Especially the BPC has the same trend of the TFP change after the year 2009, this told us that the innovation effect is inadequate, which is not good for the medical system in China, not only the innovation in providing medical services also in the medical waste disposal. For the three regions, only Eastern China had more than half of the TFP change with values greater than 1, other two regions had less than half of the TFP change with values greater than 1. This means that there is still room for TFP improvement and development in most provinces of western and central region. In the study of Liu et al. [45], they also found that the average TFP change of rural China is below 1, and the growth rate of the average TFP is − 5.1%, suggesting a downward trend with only 3 years showing an increased TFP during 2007–2016. While in the study of Wang et al. [3], they found that TFP change of China and its three regions increased from 2012 to 2015, and all the three regions in China had more than half of the TFP change with values bigger than 1. In study of Liu et al. [45], the results showed that the efficiency and TFP change values exhibited unstable trends over time. Although the EC of China and Eastern and Central regions showed an increasing trend, the western region had a declining trend. Except for central region, TGC of other two regions and China had a slight increasing trend. In addition, the BPC of both three regions and China all had a declining trend. Therefore, the BPC is the mainly obstacle for the productivity growth of health system in China and three regions, this means the lack of the innovation effect made the decline trend of TFP change of health system in China and three regions. Besides, the western regions had weak catch-up effect of health system, and the central region had weak leading effect of health system. These three effects were the contributor to productivity changes either in China or in three regions. The results of this study are less similar to those of other studies, because this paper considered the technical heterogeneity and undesirable environmental output that were not considered in those existing studies. For example, Wang et al. [3] founded that the EC of China and its three regions showed a declining trend. In Leng et al. [49] study, the TFP change during the period of 2009–2015 was greater than that during 2006–2009, which is benefited from the new round health care reform, but this policy effect was not sustainable. To analyze the efficiency of primary health care (PHC) in China, Zhang et al. [38] found the TFP had decreased by 0.6% from 2012 to 2016.

In this study, which considers environmental constraints, the efficiency of medical technology in China shows improvement year by year, which is similar to the results of existing studies [50] which do not consider environmental constraints. The rate of technological change shows a slow decline, especially after the new medical reforms, which is the same as the trend for TFP. The rate of change in technological efficiency is slowly increasing, which shows that the rate of technological change significantly determines the developmental trend of TFP changes. In other words, there is a synergistic effect between the two. Using province-level panel data from 2002 to 2008, Hu et al. [41] found that hospital efficiency was moderate when the undesirable output was adjusted for risk. They also found that, without considering undesirable outputs, the average efficiency score was overestimated and the efficiency ranking across provinces changed greatly. Finally, they found that the initiation of the New Rural Cooperative Medical System has enhanced hospital efficiency in China, especially in the developing regions.

In the context of a shortage of global medical resources relative to people’s health needs, the optimal efficiency of health resources can only be achieved through innovation and technological revolution. Technological change is the “double-edged sword”. Advances in medical technology helps effectively deal with major diseases and infectious diseases and infections, reduce pain, and shorten the length of inpatient treatment, effectively improving the efficiency of health services. Meanwhile, it cannot be ignored that, while advanced medical technology can cure sickness, medical waste from using technologies such as disposable medical devices and consumables can cause serious environmental pollution even after treatment. Therefore, to continue TFP’s growth trend in the provision of medical services in China, it is necessary to strengthen research and development for technologies to be used in medical waste treatment. This will ensure that post-treatment medical waste is released into the environment without causing environmental harm. A fundamental driver behind the growth of TFP in the provision of medical services has been an increase in desirable output paired with a reduction in waste.

The hospitals in this study include general hospitals, traditional Chinese medicine hospitals, hospitals of traditional Chinese and Western medicine, ethnic hospitals, specialized hospitals, nursing homes, and township health centers. While in China the healthcare system not only include types mentioned above, but also community health service centers, village clinics, as well as some other infirmary institutions. One reason we did not choose the latter hospitals is the afore mentioned hospitals are the main body of the healthcare system, the other reason is the data of community health service centers and village clinics is not easy to get complete data.

According to the fourth fascicle “Hospital Pollutant Generation, Emission Coefficient Manual” of the “First National Pollution Source Survey of Urban Living Sources Sewage Coefficient Manual”, the main kinds of pollutants produced by hospitals are medical waste and sewage. Therefore, these two types of pollutants are mainly considered in this paper. In fact, the environmental pollution caused by the provision of medical services is beyond air pollution and water pollution. Therefore, if all types of pollutants can be considered, the results might be more accurate.

There is another limitation of this paper is combining all hospitals defined in this paper as a whole, to analysis its meta-frontier productivity index with undesired outputs, actually this simple way of data processing will lead to some errors. It is obvious that different type hospitals, their output has extent different depending on the purpose they pursued. Thus, it is rational to consider the meta-frontier not only of regional difference, but also the type difference of medical institutions.

This study contributes to the body of existing literature in three aspects. First, it considers the environmental pollutants coming from the disposure and discharge of medical waste as an undesirable output. This undesirable output is included in the computation of the TFP. Second, it introduces the Meta-frontier method to analyze the technical heterogeneity of the medical service system in different regions. Thus, the calculation of TFP is in consideration of the heterogeneity of production outputs. Third, it uses the super efficiency non-radial SBM model, so that research objects that are DEA efficient can be sorted. For the improvement of efficiency, this study considers slack improvement in addition to input (output) improvement to ensure efficiency measurement results are as accurate as possible.

## Implications

The results indicated that, given the heterogeneity of production technology across all three regions, the TFP of China’s medical services derived from the Meta-frontier demonstrates a gradually declining trend. Such is due primarily to a lack of innovation. The decline in TFP may be alleviated by strengthening the application of technology in terms of inputs. The effect of the technology catch-up shows that the frontier of regional production technology in China is converging with the national Meta-frontier. The catch-up effect of China’s three regions has increased significantly, while the effect of innovation has shown a downward trend. The central region is in a leading trend in terms of technology, which has resulted in the leading effect gradually becoming weaker. The eastern and central regions are gradually moving closer to the Meta-frontier, with the eastern region converging faster than the central region. Province-wise, there are inter-provincial differences in the TFP changes of medical services and their decomposition. However, the technology catch-up and innovation effects in the eastern and central regions are greater than that in the western region. In many provinces in the western region, TFP changes and decomposition show negative growth.

Under existing environmental constraints, relevant government departments should improve technical innovation in the supply of healthcare services and medical waste treatment, increase technical efficiency in the factor of healthcare production, strengthen regional health planning, and balance the development of regional healthcare. For the healthcare system, the determinants of the efficiency not only include healthcare inputs, good outputs and bad outputs, but also the process management of the whole healthcare services provision. Thus, continued research is warranted.

## Data Availability

The datasets during and/or analysed during the current study available from the corresponding author on reasonable request.

## Abbreviations

TFP: Total factor productivity
SBM: Slack-based measure
GML: Global-Malmquist-Luenberger
DMUs: Decision-making units
DEA: Data envelope analysis
SFA: Stochastic frontier analysis
RSR: Rank sum ratio
TOPSIS: Technique for order preference
M: Malmquist
TE: Technological efficiency
TC: Technological change
EC: Efficiency change
TGR: Technology gap ratio

## Footnotes

1 As China has been accounting for the fixed assets of medical and health institutions since 2005, 2005–2016 was chosen as the research period, with the corresponding yearbook year being 2006–2017.

2 The eastern region includes 11 provinces and cities: Beijing, Tianjin, Hebei, Liaoning, Shanghai, Jiangsu, Zhejiang, Fujian, Shandong, Guangdong, and Hainan. The western region includes 12 provinces and cities: Inner Mongolia, Guangxi, Chongqing, Sichuan, Guizhou, Yunnan, Tibet, Shaanxi, Gansu, Qinghai, Ningxia, and Xinjiang. The central region includes 8 provinces and cities: Shanxi, Jilin, Heilongjiang, Anhui, Jiangxi, Henan, Hubei, and Hunan.

3 The hospitals in this study include general hospitals, traditional Chinese medicine hospitals, hospitals of traditional Chinese and Western medicine, ethnic hospitals, specialized hospitals, nursing homes, and township health centers.

4 The environmental pollution caused by the provision of medical services is beyond air pollution and water pollution. However, these types of pollution are perceived to be the most important ones, which have a higher availability of data than others. Moreover, according to the “Hospital Pollutant Generation, Emission Coefficient Manual” of the “First National Pollution Source Survey of Urban Living Sources Sewage Coefficient Manual”, the main kinds of pollutants produced by hospitals are medical waste and sewage. Therefore, these two types of pollutants are of primary concern in this study.

5 The specific calculation formula is not detailed here. Details can be found in the “2006 IPCC National Greenhouse Gas Inventory Guide” and “Hospital Pollutant Generation, Emission Coefficient Manual” of the “First National Pollution Source Survey of Urban Livelihood Emission Coefficient Manual”.

6 Some of the literature also refers to is as the intertemporal frontier of a group or a cluster of groups. It refers to the envelope line at the forefront of the current technology of a group of all periods; thus, it is correct to use the forefront of the global production technology of groups to express it.

## References

[1] Hu A, Yan Y, Tang X. Green development. In: Hu A, Yan Y, Tang X, editors. Xi Jinping's new development philosophy: Singapore, Springer Singapore; 2018. p. 59–71. 10.1007/978-981-10-7736-4_4.

[2] Wang D. Pormoting green transformation of medical model. Health News, 2016-02-29(006). [In Chinese] Available from: http://szb.jkb.com.cn/jkbpaper/html/2016-02/29/node_7.htm. Accessed 18 July 2020.

[3] Wang M-l, H-q F, H-b T (2017). Bootstrapping data envelopment analysis of efficiency and productivity of county public hospitals in eastern, central, and Western China after the public hospital reform. Curr Med Sci.

[4] Awodele O, Adewoye AA, Oparah AC (2016). Assessment of medical waste management in seven hospitals in Lagos, Nigeria. BMC Public Health.

[5] Adnane MI, Beklacem K, Abdelkarim E, Mohamed B (2013). Medical waste management: a case study of the Souss-Massa-Drâa region, Morocco. J Environ Prot.

[6] Patience Aseweh Abor (2007). Medical waste management at Tyberberg hospital in the Western cape South Africa.

[7] Dehghani MH, Azam K, Changani F, Dehghani EF (2008). Assessment of medical waste management in Educational Hospitals of Tehran University Medical Science. Iran J Environ Health Sci Eng.

[8] Hossain MS, Santhanam A, Nik Norulaini NA, Omar AK (2011). Clinical solid waste management practices and its impact on human health and environment. Waste Manag.

[9] Patwary MA, O’Hare WT, Street G, Elahi KM, Hossain SS, Sarke MH (2009). Country report: quantitative assessment of medical waste generation in the Capital City of Bangladesh. Waste Manag.

[10] Tamplin SA, Davidson D, Powis B, O’Leary Z (2005). Issues and option for the safe destruction and disposal of used injection materials. Waste Manag.

[11] Ma Y, Lin X, Wu A, Huang Q, Li X, Yan J. Suggested guidelines for emergency treatment of medical waste during COVID-19: Chinese experience. Waste Dispos Sustain Energy. Published 03 June 2020: doi: 10.1007/s42768-020-00039-8. 10.1007/s42768-020-00039-8 PMC7268581 32838200

[12] Jiang X, Li Y, Yan J. Hazardous waste incineration in a rotary kiln: a review. Waste Dispos Sustain Energy. 2019; 1:3–37. doi: 10.1007/s42768-019-00001-3.

[13] Jiang S, Min R, Fang P-Q (2017). The impact of healthcare reform on the efficiency of public county hospitals in China. BMC Health Serv Res.

[14] Goyal J, Singh M, Singh R, Aggarwal A (2019). Efficiency and technology gaps in Indian banking sector: Application of meta-frontier directional distance function DEA approach. J Financ Data Sci.

[15] Zhang N, Choi Y. Total-factor carbon emission performance of fossil fuel power plants in China: A metafrontier non-radial Malmquist index analysis. Energ Econ. 2013:40, 549–559. 10.1016/j.eneco.2013.08.012.

[16] Cantor VJM, Poh KL (2017). Integrated analysis of healthcare efficiency: a systematic review. J Med Syst.

[17] Bogetoft P, Otto L (2011). Benchmarking with DEA, SFA, and R.

[18] Wu J, Sun J, Song M, Liang L (2013). A ranking method for DMUs with interval data based on dea cross-efficiency evaluation and TOPSIS. J Syst Sci Syst Eng.

[19] Zhao L, Guo Y, Zhang Y, Liu F (2017). Study on efficiency of medical and health institutions at home and abroad based on data envelopment analysis. Soft Sci Health.

[20] Sherman H. Measurement of hospital technical efficiency: a comparative evaluation of data envelopment analysis and other approaches for locating inefficiency in health care organizations. United States: PhD Thesis, Harvard University; 1981.

[21] Jola-Sanchez AF, Pedraza-Martinez AJ, Bretthauer KM, Britto RA (2016). Effect of armed conflicts on humanitarian operations: Total factor productivity and efficiency of rural hospitals. J Oper Manag.

[22] Zhang X, Tone K, Lu Y (2018). Impact of the local public hospital reform on the efficiency of medium-sized hospitals in Japan: an improved slacks-based measure data envelopment analysis approach. Health Serv Res.

[23] Li N-N, Wang C-H, Ni H, Wang H (2017). Efficiency and productivity of county-level public hospitals based on the data envelopment analysis model and Malmquist index in Anhui, China. Chin Med J (Engl).

[24] Ravangard R, Hatam N, Teimourizad A, Jafari A (2014). Factors affecting the technical efficiency of health systems: A case study of Economic Cooperation Organization (ECO) countries (2004–10). Int J Health Policy Manag.

[25] Ozgen H, Ozcan YA (2002). A national study of efficiency for dialysis centers: an examination of market competition and facility characteristics for production of multiple dialysis outputs. Health Serv Res.

[26] Chilingerian JA, David SH (1997). DEA and primary care physician report cards: deriving preferred practice cones from managed care service concepts and operating strategies. Ann Oper Res.

[27] Du J, Wang J, Chen Y, Chou S-Y, Zhu J (2014). Incorporating health outcomes in Pennsylvania hospital efficiency: an additive super-efficiency DEA approach. Ann Oper Res.

[28] Chilingerian JA, Sherman HD, Cooper WW, Seiford LM, Zhu J (2011). Health-care applications: from hospitals to physicians, from productive efficiency to quality Frontiers. Handbook on data envelopment analysis.

[29] Kounetas K, Papathanassopoulos F (2013). How efficient are Greek hospitals? A case study using a double bootstrap DEA approach. Eur J Health Econ.

[30] Ni Luasa S, Dineen D, Zieba M (2018). Technical and scale efficiency in public and private Irish nursing homes – a bootstrap DEA approach. Health care Manag Sci.

[31] Costantino N, Dotoli M, Epicoco N, Falagario M, Sciancalepore F, Editors. Using cross-efficiency fuzzy data envelopment analysis for healthcare facilities performance evaluation under uncertainty. 2013 IEEE international conference on systems, man, and cybernetics; 2013 13-16 Oct. 2013. doi: 10.1109/SMC.2013.160.

[32] Chilingerian JA, David SH (1996). Benchmarking physician practice patterns with DEA: a multi-stage approach for cost containment. Ann Oper Res.

[33] O'Neill L (1998). Multifactor efficiency in Data Envelopment Analysis with an application to urban hospitals. Health Care Manag Sci.

[34] Xenos P, Yfantopoulos J, Nektarios M, Polyzos N, Tinios P, Constantopoulos A (2017). Efficiency and productivity assessment of public hospitals in Greece during the crisis period 2009–2012. Cost Effect Resour A.

[35] Mujasi PN, Asbu EZ, Puigjunoy J (2016). How efficient are referral hospitals in Uganda? A data envelopment analysis and tobit regression approach [J]. BMC Health Serv Res.

[36] Samsudin S, Jaafar AS, Applanaidu SD, Ali J, Majid R (2016). Are public hospitals in Malaysia efficient? An application of DEA and Tobit analysis. Southeast Asian J Econ.

[37] Guo H, Zhao Y, Niu T, Tsui K-L (2017). Hong Kong Hospital Authority resource efficiency evaluation: via a novel DEA-Malmquist model and Tobit regression model. Plos One.

[38] Zhang Y, Wang Q, Jiang T, Wang J (2018). Equity and efficiency of primary health care resource allocation in mainland China. Int J Equity Health.

[39] Xu X, Zhou L, Antwi HA, Chen X (2018). Evaluation of health resource utilization efficiency in community health centers of Jiangsu Province, China. Hum Resour Health.

[40] Wang X, Luo H, Qin X, Feng J, Gao H, Feng Q (2016). Evaluation of performance and impacts of maternal and child health hospital services using data envelopment analysis in Guangxi Zhuang autonomous region, China: a comparison study among poverty and non-poverty county level hospitals. Int J Equity Health.

[41] Hu H-H, Qi Q, Yang C-H. Evaluation of China's regional hospital efficiency: DEA approach with undesirable output. J Oper Res Soc. 2012;(6):63, 715–725. 10.1057/jors.2011.77.

[42] Färe R, Grosskopf S, Lindgren B, Roos P (1992). Productivity changes in Swedish pharamacies 1980–1989: a non-parametric Malmquist approach. J Prod Anal.

[43] Chung YH, Färe R, Grosskopf S (1997). Productivity and Undesirable Outputs: A Directional Distance Function Approach. J Environ Manag.

[44] D-h O (2010). A global Malmquist-Luenberger productivity index. J Prod Anal.

[45] Liu WL, Xia Y, Hou JL (2019). Health expenditure efficiency in rural China using the super-SBM model and the Malmquist productivity index. Int J Equity Health.

[46] Li H, Dong S, Liu T (2014). Relative efficiency and productivity: a preliminary exploration of public hospitals in Beijing, China. BMC Health Serv Res.

[47] Sun J, Luo HY (2017). Evaluation on equality and efficiency of health resources allocation and health services utilization in China. Int J Equity Health.

[48] Ding JM, Hu XJ, Zhang XZ, Shang L, Yu M, Chen HL (2018). Equity and efficiency of medical service systems at the provincial level of China’s mainland: a comparative study from 2009 to 2014. BMC Public Health.

[49] Leng Y, Liu WW, Xiao NZ, Li YN, Deng J (2019). The impact of policy on the intangible service efficiency of the primary health care institution-based on China’s health care reform policy in 2009. Int J Equity Health.

[50] Wang X, Cui Y, Feng R, Li J, Qian Q, Li M (2015). Efficiency characteristics and change of county hospitals. Chinese J Health Policy.

